# Characterization of Permeability Barrier Dysfunction in a Murine Model of Cutaneous Field Cancerization Following Chronic UV-B Irradiation: Implications for the Pathogenesis of Skin Cancer

**DOI:** 10.3390/cancers13163935

**Published:** 2021-08-04

**Authors:** Juan Luis Santiago, Jose Ramon Muñoz-Rodriguez, Miguel Angel de la Cruz-Morcillo, Clara Villar-Rodriguez, Lucia Gonzalez-Lopez, Carolina Aguado, Miriam Nuncia-Cantarero, Francisco Javier Redondo-Calvo, Jose Manuel Perez-Ortiz, Eva Maria Galan-Moya

**Affiliations:** 1 Department of Dermatology, University General Hospital, 13004 Ciudad Real, Spain; jlsantiago@sescam.jccm.es; 2 Translational Research Unit, University General Hospital, 13004 Ciudad Real, Spain; jmunozrodriguez@sescam.jccm.es (J.R.M.-R.); migdela@sescam.jccm.es (M.A.d.l.C.-M.); cvillarr@sescam.jccm.es (C.V.-R.); 3 Faculty of Medicine, Universidad de Castilla-La Mancha, 13071 Ciudad Real, Spain; lgonzalezl@sescam.jccm.es; 4 Department of Pathological Anatomy, University General Hospital, 13004 Ciudad Real, Spain; 5 Synaptic Structure Laboratory, Instituto de Investigación en Discapacidades Neurológicas (IDINE), Universidad de Castilla-La Mancha, 02008 Albacete, Spain; carolina.aguado@uclm.es; 6 Translational Oncology Laboratory, Centro Regional de Investigaciones Biomédicas (CRIB), Universidad de Castilla-La Mancha, 02008 Albacete, Spain; miriam.nuncia@alu.uclm.es (M.N.-C.); evamaria.galan@uclm.es (E.M.G.-M.); 7 Faculty of Nursing, Universidad de Castilla-La Mancha, 02006 Albacete, Spain

**Keywords:** cutaneous field cancerization, skin cancer, actinic keratosis, UV radiation, permeability barrier, epidermis, keratinocytes, cell proliferation, p53

## Abstract

**Simple Summary:**

In the present work, we developed an experimental preclinical model of skin with cutaneous field cancerization after chronic UV-B light exposure in an immunologically intact mouse model (SKH1 aged mice). We observed impairments in the transepidermal water loss, stratum corneum hydration, and surface pH. We also detected a marked hyperkeratotic hyperplasia of the epidermis, induction of keratinocyte hyperproliferation, incidental actinic keratosis, and in situ squamous cell carcinomas in the UV-B light-irradiated groups. In this context, the association between the permeability barrier impairment and keratinocyte hyperproliferation might be considered a new target in the management of skin with cutaneous field cancerization. As current therapeutic approaches to actinic keratosis and cutaneous field cancerization only focus on the direct antineoplastic, immunomodulatory, or photodynamic effects of approved topical drugs, this mouse model of skin with cutaneous field cancerization might be helpful for both the identification and screening of potentially new preventive strategies or treatments (e.g., skin barrier therapies).

**Abstract:**

Chronic ultraviolet B (UV-B) irradiation is known to be one of the most important hazards acting on the skin and poses a risk of developing photoaging, skin with cutaneous field cancerization (CFC), actinic keratosis (AKs), and squamous cell carcinomas (SCCs). Most of the UV-B light is absorbed in the epidermis, affecting the outermost cell layers, the stratum corneum, and the stratum granulosum, which protects against this radiation and tries to maintain the permeability barrier. In the present work, we show an impairment in the transepidermal water loss, stratum corneum hydration, and surface pH after chronic UV-B light exposure in an immunologically intact mouse model (SKH1 aged mice) of skin with CFC. Macroscopic lesions of AKs and SCCs may develop synchronically or over time on the same cutaneous surface due to both the presence of subclinical AKs and in situ SCC, but also the accumulation of different mutations in keratinocytes. Focusing on skin with CFC, yet without the pathological criteria of AKs or SCC, the presence of p53 immunopositive patches (PIPs) within the epidermis is associated with these UV-B-induced mutations. Reactive epidermis to chronic UV-B exposure correlated with a marked hyperkeratotic hyperplasia, hypergranulosis, and induction of keratinocyte hyperproliferation, while expressing an upregulation of filaggrin, loricrin, and involucrin immunostaining. However, incidental AKs and in situ SCC might show neither hypergranulosis nor upregulation of differentiation markers in the upper epidermis. Despite the overexpression of filaggrin, loricrin, involucrin, lipid enzymes, and ATP-binding cassette subfamily A member 12 (ABCA12) after chronic UV-B irradiation, the permeability barrier, stratum corneum hydration, and surface pH were severely compromised in the skin with CFC. We interpret these results as an attempt to restore the permeability barrier homeostasis by the reactive epidermis, which fails due to ultrastructural losses in stratum corneum integrity, higher pH on skin surface, abundant mast cells in the dermis, and the common presence of incidental AKs and in situ SCC. As far as we know, this is the first time that the permeability barrier has been studied in the skin with CFC in a murine model of SCC induced after chronic UV-B irradiation at high doses. The impairment in the permeability barrier and the consequent keratinocyte hyperproliferation in the skin of CFC might play a role in the physiopathology of AKs and SCCs.

## 1. Introduction

The skin is constantly exposed to external damage, such as ultraviolet A (UV-A) and ultraviolet B (UV-B) radiation. Due to their different wavelength ranges, UV-A (320–400 nm) and UV-B (280–320 nm) light act on two different levels of the skin, predominantly damaging different tissue layers. UV-A light mainly affects the dermis, whereas UV-B light acts more superficially in the epidermis [1]. Hence, the epidermis protects the body from UV-B radiation, but also from other outside hazards such as pathogenic microorganisms [2], toxic chemicals [3], allergens [4], mechanical insults [5], etc. In addition to this antimicrobial, chemical, and physical barrier, the skin must also prevent both water and electrolyte loss from the internal milieu, which is defined as the permeability barrier. In fact, the regulation of water release from the organism into the atmosphere, known as transepidermal water loss (TEWL), is the main skin function because it is crucial to maintaining the homeostasis of the internal milieu [6,7,8]. This permeability barrier is constituted by extracellular lipid membranes (extracellular lamellar bilayers), containing cholesterol, free fatty acids, and ceramides [7], and structural proteins, such as filaggrin, loricrin, and involucrin [9], located at the stratum corneum. Hence, TEWL and surface pH are widely used as indicators of the functional integrity of the stratum corneum, which is the most external part of the epidermis [6,10]. On the other hand, chronic UV-B light exposure is the main modifiable factor for skin aging [11,12]. Interestingly, both conditions, age [10,13] and chronic UV-B light exposure [14,15], show permeability barrier derangements.

Although chronic exposure to both UV-A and UV-B radiation induces a variety of harmful effects on human skin, such as epidermal thickening, collagen damage, photoaging, and skin cancer, UV-B light is considered the main cause of nonmelanoma skin cancer [12,15]. High doses of UV-B light may cause cell death (apoptosis) or induce cyclobutene pyrimidine dimers and pyrimidine(6-4)pyrimidone photoproducts in the DNA of keratinocytes, leading to mutations that may eventually result in nonmelanoma skin cancer when nucleotide excision repair fails [11,12]. As UV-B radiation acts over large areas of the skin surface, such as the scalp or the face, clinically, it is common to find patients with several lesions in those areas, such as actinic keratoses (AKs) and/or squamous cell carcinomas (SCCs) [16,17]. These lesions may be developed concomitantly or over time on the same skin surface [12,16,17]. Moreover, novel diagnostic tools such as reflectance confocal microscopy [18], optical coherence tomography [19], or in vivo fluorescent diagnosis based on protoporphyrin IX accumulation [20] may detect subclinical AKs in the same area, which are considered precursor lesions in the continuum of SCC. According to these clinical and histopathological features, the concept of cutaneous field cancerization (CFC) has been proposed to explain not only AKs within the same skin surface but also, thanks to previous diagnostic techniques, the presence of incidental AKs on the same sun-exposed skin [16,18]. This is supported by molecular changes, mainly p53 immunopositive patches (PIPs), which are detected within the epidermis in the cutaneous areas exposed to chronic UV-B radiation [11,12]. In this work, using immunocompetent mice, we established an in vivo model of chronic UV-B radiation to characterize the permeability barrier, keratinocyte hyperproliferation, and PIPs. The animals developed lesions in the continuum of photodamaged skin-AK-SCC, according to the naked eye and dermoscopic examination by a dermatologist, and displayed histopathological criteria of skin with CFC, defined as clinical and subclinical lesions (microscopic AKs, microscopic in situ SCCs, or PIPs without histopathological criteria of previous lesions within the epidermis). The main histopathological features in the epidermal architecture, such as keratinocyte hyperproliferation (acanthosis) and altered differentiation (hyperkeratosis), keratinocyte atypia, and, finally, molecular changes, such as an overexpression in the proliferating cell nuclear antigen (PCNA) and PIPs within the epidermis, were investigated by a pathologist. As PIPs in sun-exposed skin show p53 mutations in 29–64% of individual studies [21,22,23], and similar mutations have been described in normal sun/UV-B-irradiated skin and SCCs in human and murine models, it is widely accepted that PIPs may represent early precursors of SCC [22,23,24]. However, it is accepted that only a small proportion of these patches may progress to SCCs [24]. On the other hand, since keratinocyte differentiation plays a key role in epidermis homeostasis to form the stratum corneum and maintain the permeability barrier, lipid enzymes, the lipid transporter ATP-binding cassette subfamily A member 12 (ABCA12), and structural proteins linked to the skin barrier function (filaggrin, loricrin, and involucrin) were also studied for in vivo mRNA and protein expression by qPCR and Western blotting (WB). Finally, phenotypic changes, such as skin thickening, a scaly appearance, cutaneous dryness, and erythema, which characterized the cutaneous surface in our mouse model of skin with CFC, were correlated with Nile red and immunohistochemical staining for lipid and differentiation markers (filaggrin, loricrin, and involucrin), respectively, in the outermost layers of the epidermis.

## 2. Results

### 2.1. Chronic UV-B Irradiation Produced an Impairment in the Permeability Barrier in Skin with CFC

**Figure 1:**
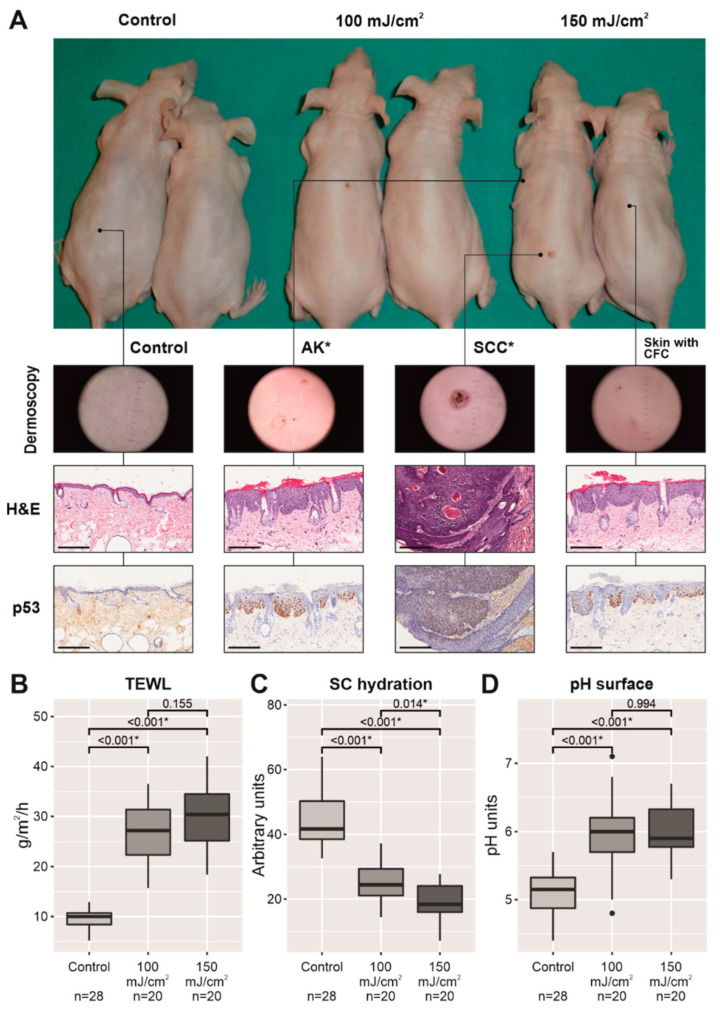
Skin appearance of two mice per group after UV-B irradiation, twice daily, 3 times per week, for 2 weeks, and then every day for another 12 weeks, from left to right: nonirradiated mice (control group), irradiated mice with 100 mJ/cm^2^ (middle), and irradiated mice with 150 mJ/cm^2^ (right). Macroscopic AKs and SCC, marked with an asterisk, were excluded from permeability, stratum corneum hydration, and surface pH measurements. The scale bar represents 200 µm (**A**). Plots with the three physiological parameters linked to the permeability barrier in nonirradiated mice (control group) and UV-B-irradiated groups with CFC (excluding skin areas with macroscopic AKs and/or SCC): TEWL values (**B**), stratum corneum hydration (**C**), and surface pH (**D**). * *p* < 0.05.

**Figure A1:**
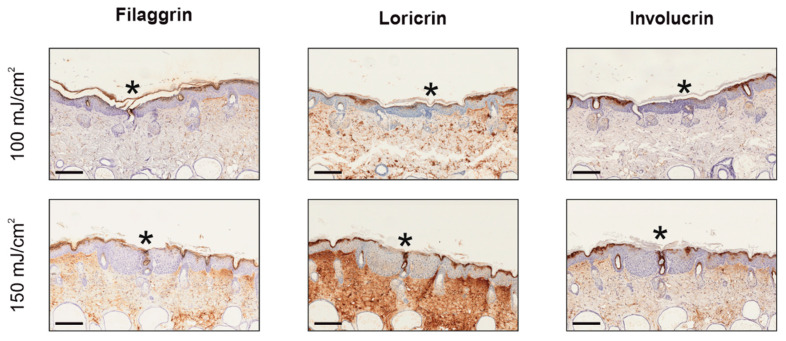
Immunostaining of incidental AKs (*) surrounded by reactive epidermis in the skin of CFC. The upregulation of filaggrin, loricrin, and involucrin is observed in the reactive epidermis (upper layers) after chronic UV-B irradiation, while incidental AKs showed lower or null immunostaining for these differentiation markers. The scale bar represents 200 µm.

**Figure A2:**
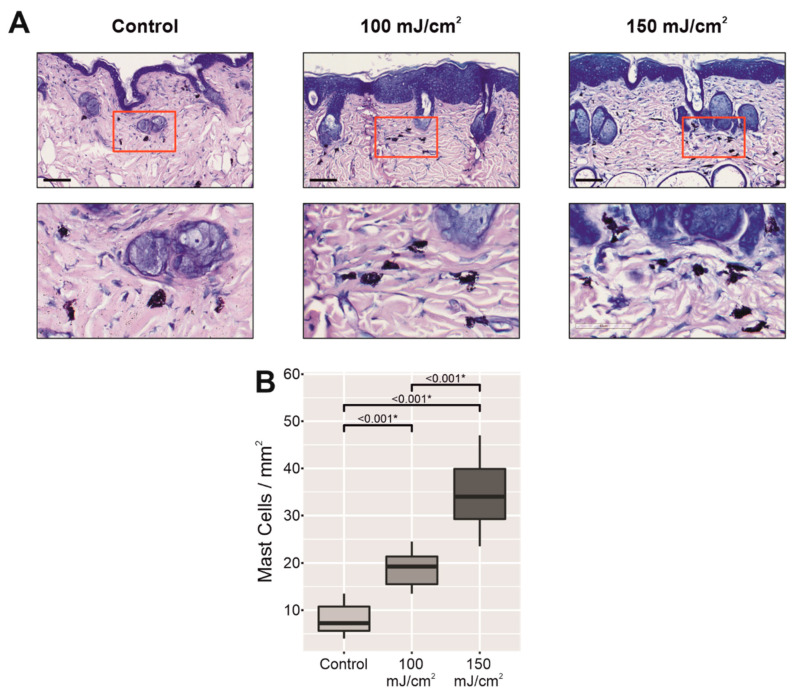
Giemsa staining to detect mast cell infiltrates in the dermis. In the bottom line, there is a zoomed-in view of the mast cells with their cytoplasm containing metachromatic granules composed of heparin and histamine. The scale bar represents 100 μm (**A**). Below, we give the quantities of mast cells in the UV-B-irradiated groups, 100 and 150 mJ/cm^2^, with respect to the nonirradiated group (**B**). Data are expressed as means ± SD of at least 10 measurements of a number of mast cells in histological samples of skin with CFC in each group with UV-B irradiated skin with respect to the nonirradiated group. * *p*-values are indicated in the figure and marked with an asterisk when significant, *p* < 0.05 (Kruskal–Wallis test with Scheffe post hoc test).

After the development of a few macroscopic AKs and/or SCCs, the back skin of the mice in both UV-B-irradiated groups showed quite homogeneous changes in appearance. A total of 21 tumors were identified as SCCs in 10 mice and 39 macroscopic AKs in 10 mice, and six tumors and 24 AKs (100 mJ/cm^2^, *n* = 5) and 15 tumors and 15 AKs (150 mJ/cm^2^, *n* = 5), were found in the irradiated skin (Table A1). In addition to macroscopic AKs and SCCs, the irradiated skin was mainly characterized by marked thickening, which masked the typical milia cysts in aged mouse skin, erythema, dryness, and scaly surface in comparison to nonirradiated skin (Figure 1A). The macroscopic features of the skin with CFC, excluding incidental AKs and in situ SCC patches (Figure A1 and Table A1), were mainly correlated with: (1) hypergranulosis with orthokeratotic acanthosis (cutaneous thickness); (2) hyperkeratosis (scaly appearance); (3) increased vascularity consisting of ectatic blood vessels in the upper dermis; and (4) abundant mast cells in the dermis (erythematous skin) (Figure A2 and Table A1). Upon examination with the naked eye, there were no clear differences between the animals in the two groups exposed to different energies of UV-B light. However, TM300 m and corneometer probes were more sensitive to changes between the groups exposed to different doses of UV-B radiation. While TEWL significantly increased in both groups exposed to chronic UV-B light (26.78 ± 1.28 g/m^2^/h, 100 mJ/cm^2^, *p* < 0.001; 29.9 ± 1.55 g/m^2^/h, 150 mJ/cm^2^, *p* < 0.001) with respect to the nonirradiated group (9.39 ± 0.38 g/m^2^/h) (Figure 1B), there was a significant decrease in stratum corneum hydration level (25.19 ± 1.24, 100 mJ/cm^2^, *p* < 0.001; 18.86 ± 1.39, 150 mJ/cm^2^, *p* < 0.001) in a dose-dependent manner with respect to the control group (43.59 ± 1.43) (Figure 1C), which correlated with the scaly surface and dryness. Moreover, the surface pH also significantly increased from its physiological acidic value (5.11 ± 0.07) to higher levels (6.00 ± 0.13, 100 mJ/cm^2^, *p* < 0.001; 6.02 ± 0.09, 150 mJ/cm^2^, *p* < 0.001) (Figure 1D). The intensity of keratinocyte atypia (keratinocyte pleomorphism and atypical mitosis), its extension within the epidermis (architectural disruption), from the basal layer to upper layers, and the number of patches of microscopic AKs and in situ SCCs increased in the UV-B-irradiated group at 150 mJ/cm^2^ in comparison to the UV-B-irradiated group at 100 mJ/cm^2^ (Table A1). Mast cell infiltrates were detected in the upper and deep dermis of the UV-B-irradiated skin, increasing in a dose-dependent manner with respect to the control group (Figure A2).

### 2.2. Chronic UV-B Irradiation Caused Epidermal Hyperplasia, PCNA Overexpression, and PIPs, Linked to the Skin with CFC

**Figure 2:**
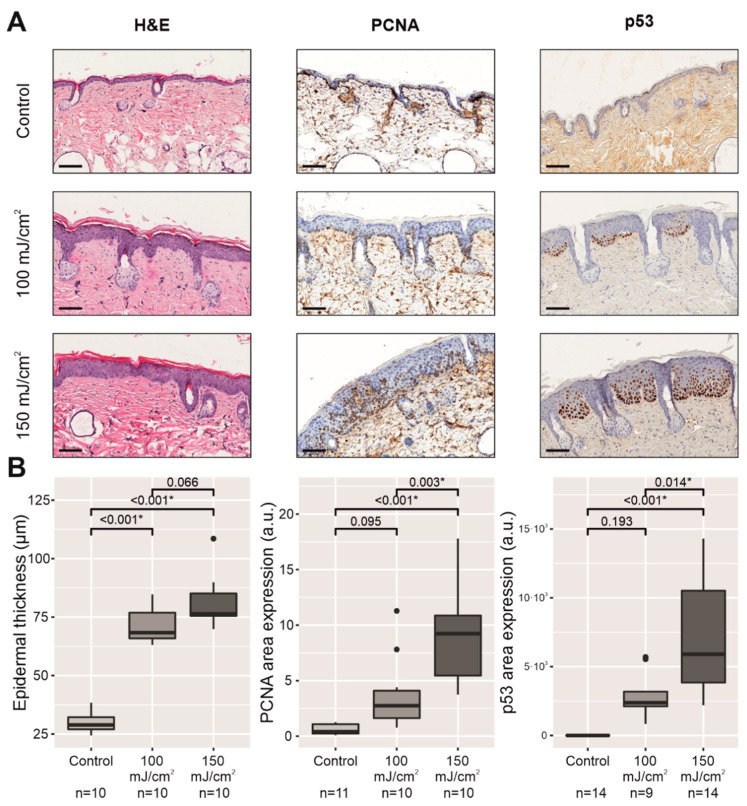
Comparative histology of nonirradiated skin with respect to UV-B-irradiated skin with CFC of the mouse back at the end of the experiment (14 weeks after initiating UV-B irradiation). The scale bar represents 100 µm (**A**). Below the histopathological images, plots represent the epidermal thickness and quantification of PCNA immunostaining and PIPs using ImageJ software and are expressed in arbitrary units (a.u.) (**B**). * *p* < 0.05.

To investigate whether chronically UV-B-irradiated skin exhibited epidermal changes with CFC according to a pathologist, homogeneous skin samples from the mouse backs were taken by a dermatologist to check the presence of patches of subclinical AKs or in situ SCCs within an epidermis with orthokeratotic acanthosis and hypergranulosis (Figure 2A, Table A1), and an overexpression in PCNA and PIPs (Figure 2A,B). AKs and in situ SCCs were characterized by parakeratotic acanthosis and the extension of the keratinocyte atypia into the upper epidermis, indicating the presence of inflammatory infiltrates in the upper dermis. The skin with CFC was defined by the presence of epidermal hyperplasia (hypergranulosis with orthokeratotic acanthosis) together with molecular markers, such as an increase in PCNA immunostaining and PIPs restricted to the lower levels of the epidermis, in the absence of keratinocyte atypia and inflammatory infiltrates. The epidermal thickness significantly increased in both UV-B-exposed groups (100 mJ/cm^2^, *p* < 0.001; 150 mJ/cm^2^, *p* < 0.001) in comparison to the control group, while PCNA immunostaining and PIPs also led to an increase in both UV-B-exposed groups, with significant changes in the 150 mJ/cm^2^ group (*p* < 0.001), in sharp contrast to the nonirradiated group (Figure 2A,B).

### 2.3. Chronic UV-B Exposure of Skin with CFC Upregulated the mRNA Expression and Protein Synthesis of Several Biomarkers Linked to the Permeability Barrier

**Figure 3:**
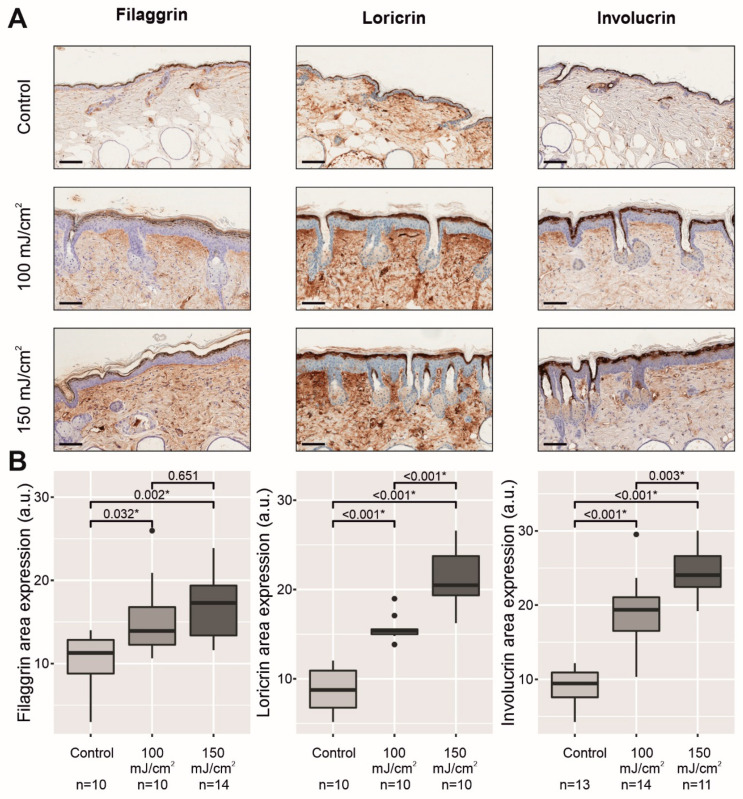
Comparative histology of nonirradiated skin with respect to UV-B-irradiated skin with CFC of the mouse back at the end of the experiment (14 weeks after initiating UV-B irradiation). From left to right, filaggrin, loricrin, and involucrin immunostaining in the upper epidermis. The scale bar represents 100 µm (**A**). Below, there is a quantitative analysis of the immunostaining for each differentiation marker using ImageJ software and expressed in arbitrary units (a.u.) (**B**). * *p* < 0.05.

Epidermal differentiation and lipid production largely determine the formation of the epidermal permeability barrier in the stratum corneum. Because there was an increase in TEWL in the apparently homogeneous skin with CFC of both groups exposed to UV-B light with respect to the nonirradiated skin in the control group (Figure 1B), we next aimed to assess whether the skin barrier disruption could be attributable to the downregulation of epidermal differentiation markers and lipid production in the outermost cell layers of the epidermis. First, immunostaining showed a significant overexpression for all three differentiation markers in the stratum corneum in both UV-B-irradiated groups compared to the nonirradiated group: filaggrin (*p* = 0.032, 100 mJ/cm^2^; *p* = 0.002, 150 mJ/cm^2^), loricrin (*p* < 0.001, 100 mJ/cm^2^; *p* < 0.001, 150 mJ/cm^2^), and involucrin (*p* < 0.001, 100 mJ/cm^2^; *p* < 0.001, 150 mJ/cm^2^) (Figure 3A,B).

**Figure 4:**
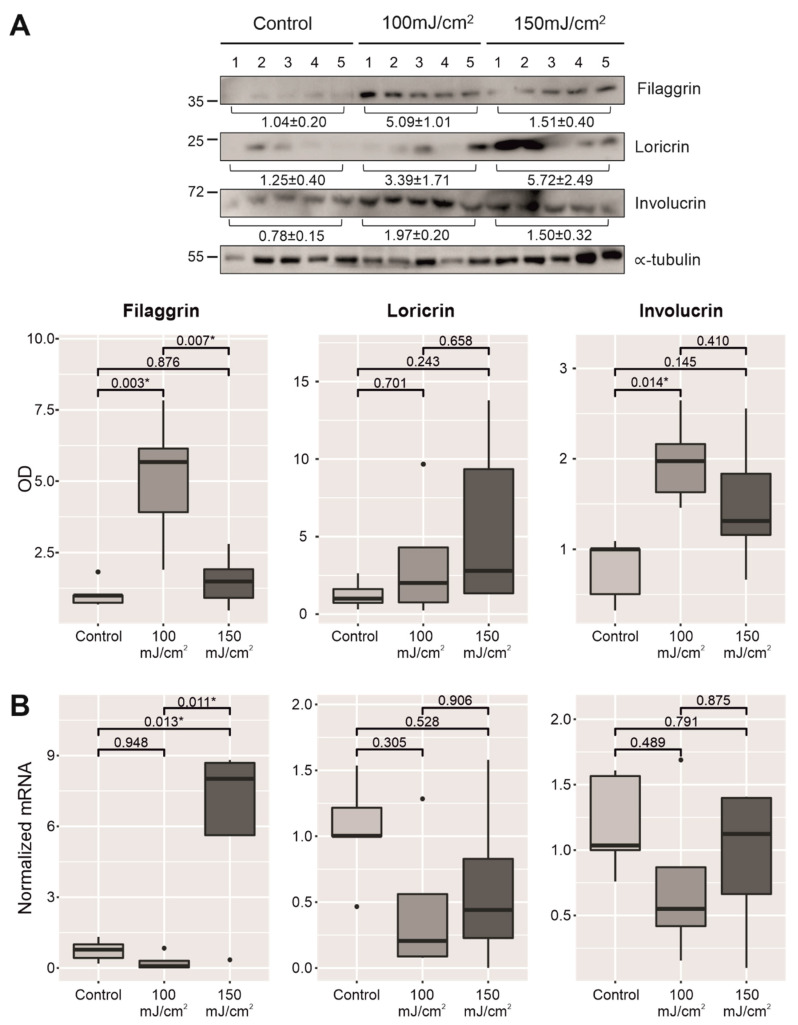
Comparative protein expression of the main differentiation markers linked to the permeability barrier (filaggrin, loricrin, and involucrin) of two groups exposed to UV-B irradiation, 100 mJ/cm^2^ and 150 mJ/cm^2^, with respect to the nonirradiated group (**A**). Below, comparative mRNA relative expression levels of these three genes in UV-B-irradiated skin, 100 mJ/cm^2^ and 150 mJ/cm^2^, with respect to the nonirradiated group (**B**). * *p* < 0.05.

The immunohistochemistry results were further confirmed through the evaluation of protein levels by WB (Figure 4A and Figure S1). Similarly, the expression of the three proteins was found to be more marked in the UV-B-irradiated groups in comparison to the control group. However, these differences at the protein level were not fully matched when the mRNA expression of the genes coding for these differentiation markers was assessed by qPCR, where only filaggrin at 150 mJ/cm^2^ showed a significant higher level (Figure 4B).

**Figure A3:**
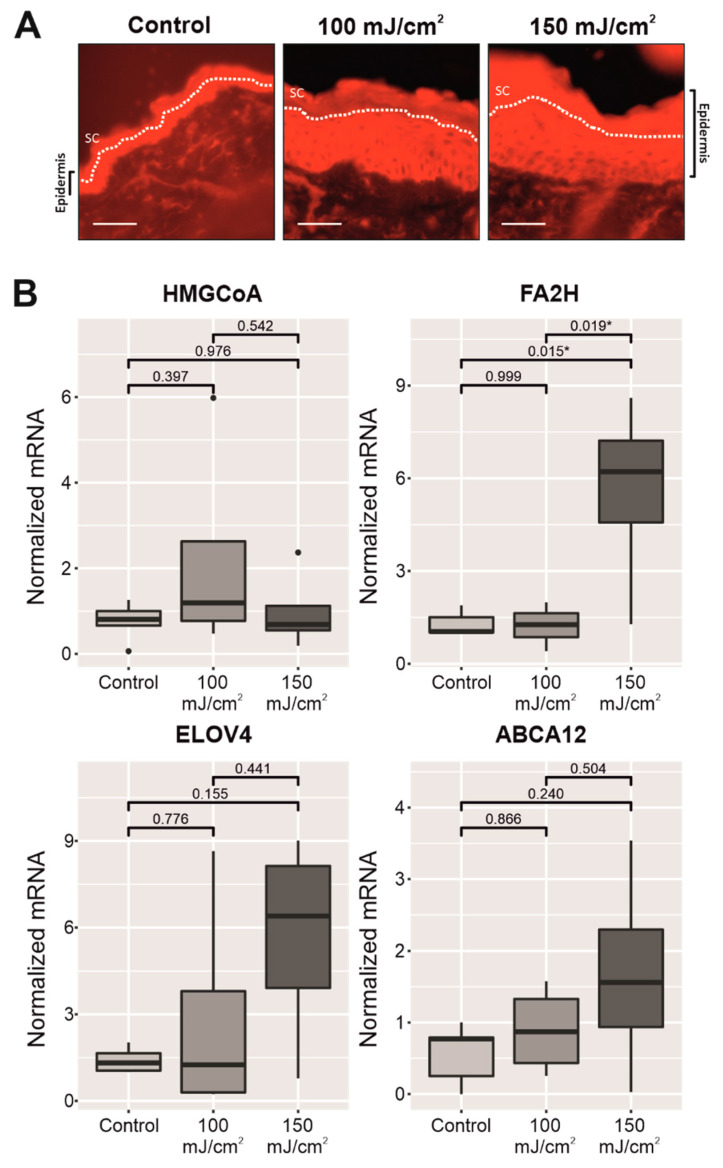
Comparative Nile red staining of skin samples from nonirradiated skin with respect to the UV-B-irradiated skin with CFC of the mouse back at the end of the experiment (14 weeks after initiating UV-B irradiation), showing more fluorescent dye uptake within the epidermis and, especially, in the outermost layers of the epidermis. SC = stratum corneum. The scale bar represents 100 μm (**A**). Below, we give the mRNA expression levels of lipid enzymes HMGCoA reductase, FA2H, ELOVL4, and lipid transporter ABCA12, linked to the permeability barrier in UV-B-irradiated skin, 100 and 150 mJ/cm^2^, with respect to the nonirradiated group (**B**). Data are expressed as means ± SD of five experiments in each group. * *p*-values are indicated in the figure and marked with an asterisk when significant, *p* < 0.05 (Kruskal–Wallis test with Scheffe post hoc test).

Next, lipid production was also investigated in the epidermis. In a first step, Nile red staining showed more lipids in the outermost layers of the hyperplastic epidermis in the irradiated skin of both UV-B-exposed groups with respect to the nonirradiated group (Figure A3). Then, we decided to evaluate the mRNA expression of key lipid enzymes (3-hydroxy-3-methyl-glutaryl-coenzyme A reductase, HMGCoA reductase; fatty acid 2-hydroxylase, FA2H; and elongation of very long chain fatty acids protein 4, ELOVL4) and a lipid transporter (ABCA12), comparing the nonirradiated group with the UV-B-irradiated groups. The in vivo mRNA expression for the lipid enzyme FA2H was significantly upregulated (*p* = 0.015) in the UV-B-irradiated group (150 mJ/cm^2^) in comparison to the nonirradiated skin (Figure A3).

## 3. Discussion

The permeability barrier depends on both the structure and chemical composition of the stratum corneum, which consists of a cell component (corneocytes) surrounded by a neutral lipid-enriched extracellular matrix (extracellular lamellar bilayers), providing a hydrophobic barrier against the movement of water and electrolytes [6,7,9]. At the ultrastructural level, this permeability barrier depends on the integrity and cohesion of both corneocytes and the extracellular lamellar bilayers of the stratum corneum. Lamellar bodies are located in the stratum granulosum, where they supply the lipids for the cornified envelopes as well as the components to form the extracellular lamellar bilayers of the stratum corneum. Several in vivo studies have investigated the UV-B-induced abnormal lamellar structures in the stratum corneum [25,26,27]. Following a single dose of UV-B light (150 mJ/cm^2^), defective lamellar bilayers were observed in the presence of an altered calcium gradient in murine epidermis. Because this altered calcium gradient may affect the secretion of lamellar body-derived lipids and enzymes to stratum corneum intercellular space, this might be an explanation for the abnormal lamellar bilayers [28].

SKH1 mice were used to establish a disease model of skin with CFC. Due to the proviral insertion of the murine leukemia virus at Hr locus, which leads to a recessive hypomorphic mutation, these mice do not develop fur but, contrary to nude mice, they are still immunocompetent [29]. According to Pillon et al., as most AKs and SCCs arise in elderly individuals who show structural differences in the skin with respect to younger individuals, SKH1 aged mice might be used to optimize their mouse model of AK [30]. Another reason to use SKH1 aged mice was because the skin barrier differs depending on age, showing an impairment in the elderly [13]. Following a previous murine model of nonmelanoma skin cancer [31], we decided to perform a chronic UV-B irradiation three times per week during the first 2 weeks. Then, we irradiated five times per week until the development of the first macroscopic lesions of SCC and AKs to accelerate the process. As soon as the first lesions were observed, we stopped irradiations and took skin samples with a homogeneous aspect: thicker, scaly, and erythematous skin. The doses of UV-B irradiation were chosen according to previous papers of chronic UV-B irradiation and carcinogenesis [32,33]. We characterized different physiological parameters of skin barrier and histopathological features of the skin with CFC (epidermal thickness, PCNA immunostaining, and PIPs overexpression). The epidermal hyperplasia and hypergranulosis were observed in the skin with CFC, which were compatible with a reactive epidermis to chronic UV-B irradiation at high doses. Pathological examination also demonstrated incidental AKs and in situ SCC surrounded by this reactive epidermis. Interestingly, in human skin with AKs and photodamage, in vivo reflectance confocal microscopy images have shown stratum corneum disruption and dermal inflammatory cells, which have been correlated with hyperkeratosis, keratinocyte atypia, and architectural disruption within the epidermis. Photodamaged skin also displayed keratinocyte atypia and architectural disruption, although this was generally less severe than in AKs, supporting the view that photodamaged skin and AKs are part of a disease continuum and photodamaged skin corresponds to CFC [34].

The skin with CFC showed different macroscopic characteristics such as erythema, scaliness, and dryness, which correlated with an increase in TEWL, lower stratum corneum hydration, and higher values of surface pH. Concurrently, most of the biomarkers linked to the permeability barrier, such as differentiation markers (filaggrin, loricrin, and involucrin), ABCA12, and two epidermal lipid enzymes (i.e., FA2H and ELOVL4), were upregulated in the epidermis of the irradiated groups. However, considering the immunostaining of differentiation markers in the upper epidermis, both incidental AKs and in situ SCC showed lower or null staining for these proteins. These immunohistopathological features were confirmed at the molecular level by WB, where an increase was observed for most of the conditions, reaching statistical significance for filaggrin and involucrin (100 mJ/cm^2^). Moreover, a significant increase in filaggrin mRNA levels at the 150 mJ/cm^2^ dose was found, a result also in line with the increase observed at the protein level in the histological studies. No significant differences were found for the rest of the conditions. One explanation for these outcomes might be the selection of areas with reactive epidermis (i.e., epidermal hyperplasia with hypergranulosis) to quantify immunostaining, while excluding skin with incidental AKs and in situ SCC according to pathological examination. However, skin samples for WB and qPCR were whole extracted (including incidental AKs and in situ SCC). In line with our histological examination, a recent study also showed a downregulation of filaggrin and other differentiation markers in AKs even at early stages [35]. On the other hand, previous studies with chronic UV-B irradiation in SKH1 mice have shown that changes in mRNA expression of other genes linked to permeability barrier depend on the moment that samples were collected [36].

The final effect of UV-B exposure in the epidermis might depend on both intensity and duration. For example, a single UV-B exposure was able to induce hyperproliferation and downregulation of filaggrin, involucrin, and loricrin expression in normal human skin grafted on to nude mice, recovering completely within 2 weeks after UV-B irradiation [37]. However, low doses of UV-B irradiation accelerated the skin barrier recovery, while increasing the expression of filaggrin and involucrin in SKH1 mice [38]. In humans, UV-B irradiation also promoted epidermal proliferation and upregulation of filaggrin, loricrin, and involucrin by immunostaining, increasing linearly according to dose and repetition [39]. Similarly to our study, chronic UV-B irradiation of SKH1 mice has been linked to an upregulation of filaggrin, loricrin, and involucrin in the upper epidermis together with an increase of TEWL in a murine model of photoaging [40,41]. To our knowledge, this is the first time that both permeability barrier and epidermal differentiation markers (filaggrin, loricrin, and involucrin) have been studied in the skin with CFC after chronic UV-B irradiation at high doses.

On the other hand, despite the impairment in the permeability barrier, a qualitative increase in the epidermal lipids in the outermost cell layers of the epidermis was observed with Nile red staining in both UV-B-irradiated groups. This feature correlated with an upregulation in the mRNA levels of ABCA12, a lipid transporter located in the lamellar bodies, and two key lipid enzymes, FA2H and ELOVL4, in the epidermis of the UV-B-irradiated groups. These two lipid enzymes are linked to the synthesis of ceramides in the stratum granulosum, which are involved in the formation of the lipid-enriched lamellar bilayers of the stratum corneum [7]. Dalmau et al., using in vitro studies, also showed an increase in involucrin and phospholipids, one of the main sources of free fatty acids in the stratum corneum, following chronic UV-B irradiation [42]. Interestingly, Löwenau et al. demonstrated that reconstructed human epidermis from UV-B-irradiated keratinocytes presented altered differentiation and impaired barrier function leading to increased permeability [43]. Although this reconstructed human epidermis from UV-B-irradiated keratinocytes displayed a thinner stratum corneum compared to normal reconstructed human epidermis, a reactive epidermis to chronic UV-B irradiation, characterized by orthokeratotic hyperplasia with hypergranulosis and PCNA and PIPs overexpression, together with several patches of incidental AKs or in situ SCCs associated with hypogranulosis were the main features in our murine model of skin with CFC. These differences in the epidermal architecture can be explained if we consider that, similarly to other published murine models of AKs and NMSC [22,30,31], our model in SKH1 mice represents a better approach to the complexity of the skin with CFC than in vitro models, including reconstructed human epidermis.

There are alternative explanations for the impairment of the permeability barrier despite an increase in several biomarkers linked to the skin barrier. Abnormal keratinocyte differentiation affects the cohesion of the stratum corneum, desquamation, permeability barrier homeostasis, and inflammation in the skin [8,44]. During normal desquamation, corneocytes detach individually from the stratum corneum in an invisible process, which involves proteolytic degradation of corneodesmosomes, disorganization of extracellular lamellar bilayers, and stratum corneum hydration [45]. The scaly appearance and dryness of the skin with CFC, including incidental AKs and in situ SCCs, which correlated with hyperkeratosis, indicate an abnormal desquamation. In fact, previous ultrastructural studies demonstrated loss in the stratum corneum integrity with respect to both of its components: corneocytes and extracellular lamellar bilayers [25,26,27]. These losses in stratum corneum integrity, which are mainly linked to the lipid structures (lamellar bilayers), might explain both the increase in TEWL and the concurrent decrease in stratum corneum hydration despite the upregulation of several biomarkers of the permeability barrier in the reactive epidermis (skin with CFC). On the other hand, higher values in surface pH might also affect the stratum corneum integrity through an increased activity of serine proteases, which degrade the corneodesmosomes, while impairing the formation of extracellular lamellar bilayers by diminishing the activity of lipid processing enzymes (β-glucocerobrosidase, sphingomyelinase, and phospholipase A2) [46,47,48]. Finally, although we did not study the changes in tight junctions in the stratum granulosum, these structures also play an important role in maintaining the permeability barrier after UV-B irradiation [49].

Like previous studies in chronically UV-B-irradiated skin in mice and humans, abundant mast cells were found in the dermis of the skin with CFC of our murine model [50,51]. Degranulation of dermal mast cells might explain why ectatic blood vessels were a common feature in the skin with CFC, correlated with its erythematous appearance [52]. Moreover, mast cells are also believed to play important roles in UV-induced immunosuppression and the development of SCCs [53,54]. Interestingly, histamine H1 and H2 receptor antagonists, topically applied, accelerate permeability barrier recovery after tape stripping and prevent epidermal hyperplasia with skin barrier disruption by acetone in murine models [55].

Hyperkeratosis, epidermal hyperplasia, and hypergranulosis due to keratinocyte hyperproliferation were the main histopathological features after chronic UV-B irradiation at high doses. Although hypogranulosis and parakeratotic acanthosis were found in incidental AKs and in situ SCCs, the most common changes in the skin with CFC were orthokeratotic acanthosis and hypergranulosis. Those features might be considered as a reactive epidermis after chronic UV-B exposure. Albibas et al. have recently reported that normal sun-exposed skin with PIPs contains multiple mutations in cancer-related genes, indicating that the clonal evolution of mutations that are detected within AKs and SCCs can also occur in PIPs within normal sun-exposed skin [56]. Because permeability barrier disruption also activates keratinocyte proliferation through epidermis-derived growth factors (amphiregulin and NGF) in an autocrine manner, this should be considered in the context of extended photodamaged areas with histopathological and molecular criteria (PCNA and PIP overexpression) of skin with CFC and incidental AKs, where clonal populations of atypical keratinocytes carrying multiple mutations in SCC-related genes might be additionally stimulated to expand within the epidermis [57,58,59]. Even though our results could potentially link permeability barrier disarrangements observed in the skin with CFC with keratinocyte hyperproliferation and mast cell infiltrates, it is important to emphasize that sustained barrier disruption alone does not suffice to produce SCC. The development of SCC requires UV-B-induced mutations in keratinocytes, its accumulation in key genes, and dysfunction of the immune system [11,12].

## 4. Materials and Methods

### 4.1. Animals

Hairless mice (Crl:SKH1-*Hr^rh^/Hr^rh^*) were obtained from Charles River Laboratories (Saint Germain-sur-L’Arbresle, France) and raised in a pathogen-free (SPF) facility with controlled temperature and humidity conditions at the Translational Research Unit of University General Hospital (Ciudad Real, Spain), until the age of 8 months and then used for all in vivo experiments. All mice were fed a standard rodent diet (Safe, Augy, France) and given tap water ad libitum. This study was reviewed and approved by the Animal Care Committee of the abovementioned hospital (Procedure ES130340000192). Animals were handled and cared for in strict accordance with the European Council and Spanish Directives (2010/63/EU, RD 53/2013), under the supervision of authorized investigators.

### 4.2. Assessment of Chronic UV-B Irradiation and Development of CFC

Hairless male SKH1 mice were kept at a 12 h light/dark cycle in the animal facility. SKH1 mice were placed in common housing (maximum: 5 mice/cage) and divided into three different experimental groups: nonirradiated control (*n* = 7), exposed to UV-B light (302 nm) at 100 mJ/cm^2^ (*n* = 5), and exposed to UV-B light (302 nm) at 150 mJ/cm^2^ (*n* = 5). The UV-B exposed groups were irradiated 3 times per week (Monday, Wednesday, and Friday) for 2 weeks, and then, every day for another 12 weeks. Eight-month-old SKH1 mice display phenotypic changes of aging (pale, thin, and dry skin with milia cysts), which is the reason why they can be considered as aged mice. After chronic UV-B irradiation, mice were 11.5 months old (normal life expectancy of hairless mice is up to 24 months).

Mice were irradiated using an Ultraviolet Crosslinker (CL-1000 model) supplied by UVP (Upland, CA, USA). The crosslinkers are designed to measure and control UV radiation within the exposure chamber. A UV sensor continually measured the UV energy and automatically adjusted to variations in UV intensity that occur when the UV tubes age. The same UV sensor was used to set UV mice exposure; it automatically deactivates the UV sources when the set UV energy dose has been achieved.

Each mouse in the UV-B-irradiated groups was examined once a week for the appearance of macroscopic AKs and/or SCCs. Growths that were >1 mm in diameter, persisted for at least 2 weeks, and showed dermoscopic features, such as hyperkeratosis, pink bumps, polypoid or verrucous growths, crusts, and atypical blood vessels, were defined as SCCs and recorded per individual and group. Macroscopic AKs were defined as tiny (less than 5 mm in size) pink, scaly lesions without atypical blood vessels during a dermoscopy assessment. The endpoint of this experiment was the development of several macroscopic AKs and/or a few SCCs (<8 mm in diameter) on the irradiated mouse back of both groups, which was defined as skin with CFC, after its evaluation with both the naked eye and dermoscopy (Heine Delta 20^®^T, Gilching, Germany) by a dermatologist. Later, these lesions were characterized by an experienced pathologist in nonmelanoma skin cancer, who also identified incidental (microscopic) AKs, in situ SCCs, and PIPs within the hyperplastic epidermis.

### 4.3. Measurement of the Skin Barrier Parameters in Skin with CFC

Transepidermal water loss (TEWL) rates, stratum corneum hydration, and surface pH on the irradiated skin of the mouse back were measured with TM300m, corneometer, and surface pH meter probes, attached to a Courage + Khazaka MPA5 system (Courage + Khazaka, Cologne, Germany). All the measurements were performed under controlled atmospheric conditions (23 °C; 60% relative humidity) after the mice developed several AKs and a few SCCs (<8 mm in diameter), which was defined as skin with CFC after UV-B irradiation (*n* = 5, 100 mJ/cm^2^; *n* = 5, 150 mJ/cm^2^). Probes were placed on four different areas on the irradiated skin of each mouse by a dermatologist, who excluded lesions of AKs and SCCs visible to the naked eye/dermoscopic examination. All skin barrier parameters were also determined in the mouse back of nonirradiated SKH1 aged mice (*n* = 7) of the same age as the two UV-B-irradiated groups when the endpoint of the postradiation skin with CFC was achieved (11.5 months old), under the same controlled atmospheric conditions, which were used as the control group.

### 4.4. Histological and Immunohistochemical Analyses

After the measurement of skin barrier parameters in the skin with CFC, UV-B-irradiated SKH1 mice were sacrificed. Biopsies (*n* = 5 per group; total = 15), 1.5 cm^2^, were excised from the irradiated skin of the mouse back (UV-B irradiated groups) or the same skin area in nonirradiated mice (control group), paraformaldehyde-fixed (24 h), and paraffin-embedded. Four-micrometer-thick sections were processed. Hematoxylin/eosin staining (DakoCytomation, Milan, Italy) was performed to measure the epidermal thickness (acanthosis with ortho- or parakeratosis) and to check keratinocyte atypia by an experienced pathologist. Giemsa staining (Merck, Darmstadt, Germany) was used to detect mast cells in the dermis. For immunohistochemistry, dewaxing and antigen retrieval were performed by immersing the slides in EnVision™ Flex Target Retrieval Solution and heating in the PT module, according to the manufacturer’s instructions. After pretreatment, the slides were incubated with the following primary antibodies for 30 min: filaggrin 1:500 (Biolegend, San Diego, CA, USA), loricrin 1:1800 (Abcam, Cambridge, UK), involucrin 1:500 (Biolegend), PCNA 1:150 (Agilent, Madrid, Spain), and p53 1:750 (CM5, Leica Pleasanton, CA, USA). The reactions were detected using the EnVision™ Flex/HRP Detection Reagent (Cat. no K8000; DakoCytomation) standard polymer technique. In addition, the signal intensity was amplified using EnVision™ Flex+ Rabbit linker (Cat. no. K8009; DakoCytomation). Finally, sections were counterstained with hematoxylin and mounted with DePex (VWR, Poole, UK). Negative controls were obtained by omitting the primary antibody. For immunostaining quantification for area expression, samples were first scanned by a computer-assisted image system (Leica Microsystems, Wetzlar, Germany) and later analyzed to quantify staining and compare samples of the two groups exposed to UV-B light with respect to the nonirradiated group using ImageJ software version 1.52a (NIH, Bethesda, MD, USA).

To localize and perform a qualitative evaluation of the lipids in the epidermis, 0.001% Nile red (Sigma-Aldrich, Darmstadt, Germany) in acetone plus 75% glycerol in deionized water was used in frozen tissue sections and then examined under an epifluorescence microscope (Nikon Eclipse 90i, Tokyo, Japan), after 5 min, 530/25 nm excitation, at 590/35 nm emission.

### 4.5. Western Immunoblotting

After skin excision and the removal of subcutaneous fat with a scalpel, samples were chosen after excluding areas with macroscopic AKs or SCCs and deposited in a Petri dish with phosphate-buffered saline. After that, the Petri dishes were placed in a water bath at 60 °C for 1 min in order to facilitate the epidermis removal from the subjacent dermis using a scalpel. Then, the epidermal fractions (*n* = 5 for each group) were processed for Western blotting. After mechanical and enzymatic disaggregation in ice-cold RIPA lysis buffer (20 mM Tris-HCl (pH 7.5), 150 mM NaCl, 1 mM Na2EDTA, 1 mM EGTA, 1% NP-40, 1% sodium deoxycholate, 2.5 mM sodium pyrophosphate, 1 mM b-glycerophosphate, 1 mM Na3VO4, 1 μg/mL leupeptin) for 30 min, samples were centrifuged at 10,000× *g* for 10 min to remove the insoluble material and purify the extract. Then, the protein concentration was determined using a bicinchoninic acid (BCA) protein assay kit (Thermo Fisher Scientific, Waltham, MA, USA). After the addition of a loading buffer (5× Thermo Fisher Scientific) and protein denaturalization, 50 µg of the total protein lysate was used to perform 10% sodium dodecyl sulfate polyacrylamide gel electrophoresis (SDS-PAGE). After electrophoresis, the proteins in gels were transferred to polyvinylidene difluoride membranes (Millipore Corporation, Bedford, MA, USA). Then, membranes were blocked (1 h) with 5% skimmed milk in Tween-Tris-buffered saline (T-TBS) (100 mM Tris (pH 7.5), 150 mM NaCl, 0.05% Tween 20). Primary human monoclonal/polyclonal antibodies against loricrin (1:500, Abcam, Cambridge, UK), filaggrin (1:500, Abcam), involucrin (1:500, Abcam), and alpha-tubulin (1:2000, Santa Cruz Biotechnology, Dallas, TX, USA) were used to incubate the membranes overnight at 4 °C. Then, after extensive washing with T-TBS, membranes were incubated with HRP-conjugated anti-mouse or anti-rabbit secondary antibodies for 1 h at room temperature, and protein bands were visualized by chemiluminescence using the ECL Plus Western Blotting Detection System (GE Healthcare, Amersham, UK) and a LAS-4000 developer (Fujifilm, Tokyo, Japan). The images obtained were quantified by performing a densitometric analysis of the different bands, using ImageJ software. The values (given as arbitrary units) were normalized by considering the total protein levels of each sample. In all cases, alpha-tubulin was used as a loading control.

### 4.6. qPCR for mRNA Expression

Total RNA was isolated from the skin of the mouse back of the UV-B-irradiated groups (100 and 150 mJ/cm^2^) and from the back of the nonirradiated group (*n* = 5 for each group) using TRI Reagent (Applied Biosystems, Madrid, Spain). RNA concentrations and purities were determined using a NanoDrop ND-1000 spectrophotometer (Thermo Fisher Scientific). First strand cDNA was synthesized from 1 µg of total RNA using the Kit Maxima First Strand cDNA Synthesis Kit (Thermo Fisher Scientific), in a thermocycler (Bio-Rad, Hercules, CA, USA), under the following reaction conditions: 37 °C for 2 min, 25 °C for 10 min, 50 °C for 15 min and 85 °C for 5 min. The resulting cDNAs were subjected to quantitative real-time PCR analysis using a Fast SYBR Green Master Mix (Thermo Fisher Scientific) in a LightCycler^®^ 480 Instrument II Real-Time PCR system (Roche, Basel, Switzerland). The conditions used included an initial step at 94 °C for 2 min, followed by 40 cycles at 94 °C for 30 s, 60 °C for 45 s, and 72 °C for 60 s. Each sample was analyzed in triplicate, and the cycle threshold (Ct) values of transcripts were determined using LightCycler^®^ 480 software version 1.5. Ct values were calculated using 18S RNA as a reference. The quantification was performed by analysis of relative gene expression data using the 2^-ΔΔCt^ method [60], with 18S RNA expression level as housekeeping gene. Primer sequences are listed in Table A2. The relative expression of the mRNAs in the UV-B-irradiated groups (100 and 150 mJ/cm^2^) were compared to the control mRNA of nonirradiated mice of the same age. Data are expressed as the fold increases over the controls.

### 4.7. Statistics

All the data are expressed as mean ± SEM and were analyzed using the SPSS software (version 24.0 for Windows, IBM, Armonk, NY, USA) and represented in plots using R 3.6.1116 (R Statistics, Vienna, Austria). A normality analysis was performed using the Shapiro–Wilk test. In all the experiments, a Kruskal–Wallis test with a Scheffe post hoc test was used to evaluate the differences between three groups. Significance was established at *p* < 0.05.

## 5. Conclusions

In the present work, we characterized the permeability barrier with respect to its physiological, histopathological, and molecular changes in an immunocompetent mouse model of skin with CFC induced upon chronic UV-B irradiation. Although changes in skin with CFC included upregulation in differentiation markers and the lipid enzymes FA2H and ELOVL4, these were unable to restore the permeability barrier. This fact might be explained by permanent losses in stratum corneum integrity at the ultrastructural level (lamellar bilayers and lamellar body secretion), which are induced by UV-B irradiation. A higher pH on the skin surface and abundant mast cells in the dermis, which also affect the stratum corneum lipids, might contribute to a global detrimental effect on the permeability barrier despite the attempt of the reactive epidermis to restore both lipids and proteins in the upper epidermis. As current therapeutic approaches to AKs and CFC only focus on direct antineoplastic, immunomodulatory, or photodynamic effects of approved topical drugs, this mouse model of skin with CFC might be helpful for the identification and screening of potential new preventive strategies or treatments (e.g., skin barrier therapies).

## Data Availability

The data presented in this study are available on request from the corresponding author.

## Supplementary Materials

The following are available online at https://www.mdpi.com/article/10.3390/cancers13163935/s1, Figure S1: Uncropped original Western Blot.

## Abbreviations

ABCA12	ATP-binding cassette subfamily A member 12
AK	Actinic keratosis
CFC	Cutaneous field cancerization
DNA	Deoxyribonucleic acid
ELOVL4	Elongation of very long chain fatty acids protein 4
FA2H	Fatty acid 2-hydroxylase
HMGCoA reductase	3-hydroxy-3-methyl-glutaryl-coenzyme A reductase
mRNA	Messenger RNA
PCNA	Proliferating cell nuclear antigen
PI3K	Phosphoinositide 3-kinase
PIP	p53 immunopositive patch
p53	Tumor suppressor p53
qPCR	Quantitative polymerase chain reaction
SCC	Squamous cell carcinoma
SEM	Standard error of the mean
TEWL	Transepidermal water loss
UV-A light	Ultraviolet A light
UV-B light	Ultraviolet B light
WB	Western blotting

## Appendix A

**Table A1:** Naked eye, dermoscopic, and histopathological features in the UV-B-irradiated groups compared to the nonirradiated group.

Group	*n*	Skin Appearance	Number of Macroscopic AKs/SCCs	Dermoscopy	Histopathology (Skin with CFC)
**Control group**	1	Aged mouse skin: thin pale skin with milia cysts.	None	Thin pale skin. Milia cysts.	Thin (normal) epidermis without keratinocyte atypia. Milia cysts in the dermis.
	2	Aged mouse skin: thin pale skin with milia cysts.	None	Thin pale skin. Milia cysts.	Thin (normal) epidermis without keratinocyte atypia. Milia cysts in the dermis.
	3	Aged mouse skin: thin pale skin with milia cysts.	None	Thin pale skin. Milia cysts.	Thin (normal) epidermis without keratinocyte atypia. Milia cysts in the dermis.
	4	Aged mouse skin: thin pale skin with milia cysts.	None	Thin pale skin. Milia cysts.	Thin (normal) epidermis without keratinocyte atypia. Milia cysts in the dermis.
	5	Aged mouse skin: thin pale skin with milia cysts.	None	Thin pale skin. Milia cysts.	Thin (normal) epidermis without keratinocyte atypia. Milia cysts in the dermis.
**UV-B** **Irradiated Skin 100 mJ/cm^2^**	1	Thicker erythematous scaly skin. Cutaneous dryness. Few hyperkeratotic verrucous lesions.	**Nº AKs**: 5 **Nº SCCs**: 0	**AKs**: Pink scaly structures without vessels.	Orthokeratotic hyperplasia of the epidermis with few patches of parakeratosis corresponding to microscopic AKs. Hypergranulosis. Keratinocyte atypia. Mast cells in the dermis. Milia cysts in the dermis.
	2	Thicker erythematous scaly skin. Cutaneous dryness. Few hyperkeratotic verrucous lesions.	**Nº AKs**: 3 **Nº SCCs**: 2	**AKs**: Pink scaly structures without vessels. **SCCs**: Scaly crusted lesions with polymorphic vessels.	Orthokeratotic hyperplasia of the epidermis with few patches of microscopic AKs. Hypergranulosis. Keratinocyte atypia. Few inflammatory infiltrates in AKs. Mast cells in the dermis. Milia cysts in the dermis.
	3	Thicker erythematous scaly skin. Cutaneous dryness. Few hyperkeratotic lesions.	**Nº AKs**: 5 **Nº SCCs**: 0	**AKs**: Pink scaly structures without vessels.	Orthokeratotic hyperplasia of the epidermis with few patches of microscopic AKs and in situ SCC. Hypergranulosis. Keratinocyte atypia. Few inflammatory infiltrates in AKs or in situ SCC. Mast cells in the dermis. Milia cysts in the dermis.
	4	Thicker erythematous scaly skin. Cutaneous dryness.	**Nº AKs**: 2 **Nº SCCs**: 0	**AKs**: Pink scaly structures without vessels.	Orthokeratotic hyperplasia of the epidermis with few patches of microscopic AKs. Hypergranulosis. Keratinocyte atypia. Few inflammatory infiltrates in AKs. Mast cells in the dermis. Ectatic blood vessels in the dermis. Milia cysts in the dermis.
	5	Thicker erythematous scaly skin. Cutaneous dryness. Few hyperkeratotic verrucous lesions.	**Nº AKs**: 9 **Nº SCCs**: 4	**AKs**: Pink scaly structures without vessels. **SCCs**: Whitish structures with polymorphic vessels.	Orthokeratotic hyperplasia of the epidermis with few patches of microscopic AKs. Hypergranulosis. Keratinocyte atypia. Few inflammatory infiltrates in some of the AKs. Ectatic blood vessels in the dermis. Milia cysts in the dermis.
**UV-B** **Irradiated Skin 150 mJ/cm^2^**	1	Thicker scaly erythematous skin. Cutaneous dryness. Hyperkeratotic verrucous lesions.	**Nº AKs**: 2 **Nº SCCs**: 6	**AKs**: Pink scaly structures without vessels. **SCCs**: Verrucous hyperkeratotic lesions, central crust, and atypical vessels. Polypoid whitish lesions with polymorphic vessels.	Orthokeratotic hyperplasia of the epidermis. Hypergranulosis. Microscopic AKs (more common). Marked keratinocyte atypia. Few inflammatory infiltrates in some of the AKs. Ectatic blood vessels in the dermis. Mast cells in the dermis. Milia cysts in the dermis.
	2	Thicker erythematous scaly skin. Cutaneous dryness. Few hyperkeratotic verrucous lesions.	**Nº AKs**: 3 **Nº SCCs**: 3	**AKs**: Pink scaly structures without vessels. **SCCs**: Crusted and whitish structures with polymorphic vessels.	Orthokeratotic hyperplasia of the epidermis. Hypergranulosis. Microscopic AKs (more common) and in situ SCC. Marked keratinocyte atypia. Few inflammatory infiltrates in some of the AKs. Mast cells in the dermis. Milia cysts in the dermis.
	3	Thicker erythematous scaly skin. Cutaneous dryness. Hyperkeratotic verrucous lesions.	**Nº AKs**: 5 **Nº SCCs**: 6	**AKs**: Pink scaly structures without vessels. **SCCs**: Hyperkeratotic whitish structures, central crust, and looped vessels. Polypoid whitish lesions with polymorphic vessels.	Orthokeratotic hyperplasia of the epidermis. Hypergranulosis. Microscopic AKs. Keratinocyte atypia. Few inflammatory infiltrates in AKs. Mast cells in the dermis. Milia cysts in the dermis.
	4	Thicker erythematous scaly skin. Cutaneous dryness.	**Nº AKs**: 3 **Nº SCCs**: 0	**AKs**: Pink scaly structures without vessels.	Orthokeratotic hyperplasia of the epidermis. Hypergranulosis. Microscopic AKs. Marked keratinocyte atypia. Few inflammatory infiltrates in AKs. Mast cells in the dermis. Ectatic blood vessels in the dermis. Milia cysts in the dermis.
	5	Thicker erythematous scaly skin. Cutaneous dryness.	**Nº AKs**: 2 **Nº SCCs**: 0	**AKs**: Pink scaly structures without vessels.	Orthokeratotic hyperplasia of the epidermis. Hypergranulosis. Microscopic AKs and in situ SCC. Marked keratinocyte atypia. Mast cells in the dermis. Ectatic blood vessels in the dermis. Milia cysts in the dermis.

**Table A2:** Primers for qRT-PCR.

Gene	Forward Primer (5′–3′)	Reverse Primer (5′–3′)
*18S RNA*	GTAACCCGTTGAACCCCATT	CCATCCAATCGGTAGTAGTAGCG
*mFilaggrin*	ATGTCCGCTCTCCTGGAAAG	TGGATTCTTCAAGACTGCCTGTA
*mLoricrin*	GTGGAAAGACCTCTGGTGGA	TGGAACCACCTCCATAGGAA
*mInvolucrin*	AAGGGCTTTCCCAAACATGA	TGCTGGTGCTCACACTTTTGA
*mHMGCoA reductase*	CTTGTGGAATGCCTTGTGAT	CCGAAGCAGCACATGATC
*mFA2H*	CGCTGGCTGGAGCAGTACTAT	TGCAGAGGCTACAGCACCATT
*mELOVL4*	CGATAAGCATAAGCACGCTCTATC	AACGGCTCGCGGTCTTTC
*mABCA12*	AGGATGGCTTCCCAGTTTCA	TGGCCATAAGATCAAGACAAGTGT

## References

[1] Haywood R., Rogge F., Lee M. (2008). Protein, Lipid, and DNA Radicals to Measure Skin UVA Damage and Modulation by Melanin. Free Radic. Biol. Med..

[2] Jinnestål C.L., Belfrage E., Bäck O., Schmidtchen A., Sonesson A. (2014). Skin Barrier Impairment Correlates with Cutaneous Staphylococcus Aureus Colonization and Sensitization to Skin-Associated Microbial Antigens in Adult Patients with Atopic Dermatitis. Int. J. Dermatol..

[3] Kim B.E., Kim J., Goleva E., Berdyshev E., Lee J., Vang K.A., Lee U.H., Han S., Leung S., Hall C.F. (2021). Particulate Matter Causes Skin Barrier Dysfunction. JCI Insight.

[4] Jakasa I., Thyssen J.P., Kezic S. (2018). The Role of Skin Barrier in Occupational Contact Dermatitis. Exp. Dermatol..

[5] Pigors M., Kiritsi D., Cobzaru C., Schwieger-Briel A., Suárez J., Faletra F., Aho H., Mäkelä L., Kern J.S., Bruckner-Tuderman L. (2012). TGM5 Mutations Impact Epidermal Differentiation in Acral Peeling Skin Syndrome. J. Investig. Dermatol..

[6] Proksch E., Brandner J.M., Jensen J.-M. (2008). The Skin: An Indispensable Barrier. Exp. Dermatol..

[7] Feingold K.R., Elias P.M. (2014). Role of Lipids in the Formation and Maintenance of the Cutaneous Permeability Barrier. Biochim. Biophys. Acta.

[8] Schmuth M., Blunder S., Dubrac S., Gruber R., Moosbrugger-Martinz V. (2015). Epidermal Barrier in Hereditary Ichthyoses, Atopic Dermatitis, and Psoriasis. J. Dtsch. Dermatol. Ges..

[9] Nemes Z., Steinert P.M. (1999). Bricks and Mortar of the Epidermal Barrier. Exp. Mol. Med..

[10] Choi E.-H., Man M.-Q., Xu P., Xin S., Liu Z., Crumrine D.A., Jiang Y.J., Fluhr J.W., Feingold K.R., Elias P.M. (2007). Stratum Corneum Acidification Is Impaired in Moderately Aged Human and Murine Skin. J. Investig. Dermatol..

[11] Ichihashi M., Ueda M., Budiyanto A., Bito T., Oka M., Fukunaga M., Tsuru K., Horikawa T. (2003). UV-Induced Skin Damage. Toxicology.

[12] Ratushny V., Gober M.D., Hick R., Ridky T.W., Seykora J.T. (2012). From Keratinocyte to Cancer: The Pathogenesis and Modeling of Cutaneous Squamous Cell Carcinoma. J. Clin. Investig..

[13] Ramos-e-Silva M., Boza J.C., Cestari T.F. (2012). Effects of Age (Neonates and Elderly) on Skin Barrier Function. Clin. Dermatol..

[14] Biniek K., Levi K., Dauskardt R.H. (2012). Solar UV Radiation Reduces the Barrier Function of Human Skin. Proc. Natl. Acad. Sci. USA.

[15] Permatasari F., Zhou B., Luo D. (2013). Epidermal Barrier: Adverse and Beneficial Changes Induced by Ultraviolet B Irradiation Depending on the Exposure Dose and Time (Review). Exp. Ther. Med..

[16] Fernandez Figueras M.T. (2017). From Actinic Keratosis to Squamous Cell Carcinoma: Pathophysiology Revisited. J. Eur. Acad. Dermatol. Venereol..

[17] Schmitz L., Oster-Schmidt C., Stockfleth E. (2018). Nonmelanoma Skin Cancer—From Actinic Keratosis to Cutaneous Squamous Cell Carcinoma. J. Dtsch. Dermatol. Ges..

[18] Ulrich M., Maltusch A., Röwert-Huber J., González S., Sterry W., Stockfleth E., Astner S. (2007). Actinic Keratoses: Non-Invasive Diagnosis for Field Cancerisation. Br. J. Dermatol..

[19] Friis K.B.E., Themstrup L., Jemec G.B.E. (2017). Optical Coherence Tomography in the Diagnosis of Actinic Keratosis-A Systematic Review. Photodiagnosis Photodyn. Ther..

[20] Tyrrell J., Campbell S.M., Curnow A. (2011). Monitoring the Accumulation and Dissipation of the Photosensitizer Protoporphyrin IX during Standard Dermatological Methyl-Aminolevulinate Photodynamic Therapy Utilizing Non-Invasive Fluorescence Imaging and Quantification. Photodiagnosis Photodyn. Ther..

[21] Jonason A.S., Kunala S., Price G.J., Restifo R.J., Spinelli H.M., Persing J.A., Leffell D.J., Tarone R.E., Brash D.E. (1996). Frequent Clones of P53-Mutated Keratinocytes in Normal Human Skin. Proc. Natl. Acad. Sci. USA.

[22] Kramata P., Lu Y.-P., Lou Y.-R., Singh R.N., Kwon S.M., Conney A.H. (2005). Patches of Mutant P53-Immunoreactive Epidermal Cells Induced by Chronic UVB Irradiation Harbor the Same P53 Mutations as Squamous Cell Carcinomas in the Skin of Hairless SKH-1 Mice. Cancer Res..

[23] Robinson S., Dixon S., August S., Diffey B., Wakamatsu K., Ito S., Friedmann P.S., Healy E. (2010). Protection against UVR Involves MC1R-Mediated Non-Pigmentary and Pigmentary Mechanisms in Vivo. J. Investig. Dermatol..

[24] Rebel H., Kram N., Westerman A., Banus S., van Kranen H.J., de Gruijl F.R. (2005). Relationship between UV-Induced Mutant P53 Patches and Skin Tumours, Analysed by Mutation Spectra and by Induction Kinetics in Various DNA-Repair-Deficient Mice. Carcinogenesis.

[25] Holleran W.M., Uchida Y., Halkier-Sorensen L., Haratake A., Hara M., Epstein J.H., Elias P.M. (1997). Structural and Biochemical Basis for the UVB-Induced Alterations in Epidermal Barrier Function. Photodermatol. Photoimmunol. Photomed..

[26] Meguro S., Arai Y., Masukawa Y., Uie K., Tokimitsu I. (2000). Relationship between Covalently Bound Ceramides and Transepidermal Water Loss (TEWL). Arch. Dermatol. Res..

[27] Takagi Y., Nakagawa H., Kondo H., Takema Y., Imokawa G. (2004). Decreased Levels of Covalently Bound Ceramide Are Associated with Ultraviolet B-Induced Perturbation of the Skin Barrier. J. Investig. Dermatol..

[28] Jiang S.J., Chu A.W., Lu Z.F., Pan M.H., Che D.F., Zhou X.J. (2007). Ultraviolet B-Induced Alterations of the Skin Barrier and Epidermal Calcium Gradient. Exp. Dermatol..

[29] Schaffer B.S., Grayson M.H., Wortham J.M., Kubicek C.B., McCleish A.T., Prajapati S.I., Nelon L.D., Brady M.M., Jung I., Hosoyama T. (2010). Immune Competency of a Hairless Mouse Strain for Improved Preclinical Studies in Genetically Engineered Mice. Mol. Cancer Ther..

[30] Pillon A., Gomes B., Vandenberghe I., Cartron V., Cèbe P., Blanchet J.-C., Sibaud V., Guilbaud N., Audoly L., Lamant L. (2017). Actinic Keratosis Modelling in Mice: A Translational Study. PLoS ONE.

[31] Cozzi S.-J., Ogbourne S.M., James C., Rebel H.G., de Gruijl F.R., Ferguson B., Gardner J., Lee T.T., Larcher T., Suhrbier A. (2012). Ingenol Mebutate Field-Directed Treatment of UVB-Damaged Skin Reduces Lesion Formation and Removes Mutant P53 Patches. J. Investig. Dermatol..

[32] Divya S.P., Wang X., Pratheeshkumar P., Son Y.-O., Roy R.V., Kim D., Dai J., Hitron J.A., Wang L., Asha P. (2015). Blackberry Extract Inhibits UVB-Induced Oxidative Damage and Inflammation through MAP Kinases and NF-ΚB Signaling Pathways in SKH-1 Mice Skin. Toxicol. Appl. Pharmacol..

[33] Huang K.M., Liang S., Yeung S., Oiyemhonlan E., Cleveland K.H., Parsa C., Orlando R., Meyskens F.L., Andresen B.T., Huang Y. (2017). Topically Applied Carvedilol Attenuates Solar Ultraviolet Radiation Induced Skin Carcinogenesis. Cancer Prev. Res..

[34] Tan J.M., Lambie D., Sinnya S., Sahebian A., Soyer H.P., Prow T.W., Ardigò M. (2016). Histopathology and Reflectance Confocal Microscopy Features of Photodamaged Skin and Actinic Keratosis. J. Eur. Acad. Dermatol. Venereol..

[35] Persechino F., Ranieri D., Guttieri L., Nanni M., Torrisi M.R., Belleudi F. (2021). Expression Profile of Fibroblast Growth Factor Receptors, Keratinocyte Differentiation Markers, and Epithelial Mesenchymal Transition-Related Genes in Actinic Keratosis: A Possible Predictive Factor for Malignant Progression?. Biology.

[36] Bak H., Hong S.-P., Jeong S.-K., Choi E.-H., Lee S.E., Lee S.-H., Ahn S.-K. (2011). Altered Epidermal Lipid Layers Induced by Long-Term Exposure to Suberythemal-Dose Ultraviolet. Int. J. Dermatol..

[37] Del Bino S., Vioux C., Rossio-Pasquier P., Jomard A., Demarchez M., Asselineau D., Bernerd F. (2004). Ultraviolet B Induces Hyperproliferation and Modification of Epidermal Differentiation in Normal Human Skin Grafted on to Nude Mice. Br. J. Dermatol..

[38] Hong S.P., Kim M.J., Jung M.-Y., Jeon H., Goo J., Ahn S.K., Lee S.H., Elias P.M., Choi E.H. (2008). Biopositive Effects of Low-Dose UVB on Epidermis: Coordinate Upregulation of Antimicrobial Peptides and Permeability Barrier Reinforcement. J. Investig. Dermatol..

[39] Lee J.H., An H.T., Chung J.H., Kim K.H., Eun H.C., Cho K.H. (2002). Acute Effects of UVB Radiation on the Proliferation and Differentiation of Keratinocytes. Photodermatol. Photoimmunol. Photomed..

[40] Kambayashi H., Yamashita M., Odake Y., Takada K., Funasaka Y., Ichihashi M. (2001). Epidermal Changes Caused by Chronic Low-Dose UV Irradiation Induce Wrinkle Formation in Hairless Mouse. J. Dermatol. Sci..

[41] Kambayashi H., Odake Y., Takada K., Funasaka Y., Ichihashi M. (2003). Involvement of Changes in Stratum Corneum Keratin in Wrinkle Formation by Chronic Ultraviolet Irradiation in Hairless Mice. Exp. Dermatol..

[42] Dalmau N., Andrieu-Abadie N., Tauler R., Bedia C. (2018). Phenotypic and Lipidomic Characterization of Primary Human Epidermal Keratinocytes Exposed to Simulated Solar UV Radiation. J. Dermatol. Sci..

[43] Löwenau L.J., Zoschke C., Brodwolf R., Volz P., Hausmann C., Wattanapitayakul S., Boreham A., Alexiev U., Schäfer-Korting M. (2017). Increased Permeability of Reconstructed Human Epidermis from UVB-Irradiated Keratinocytes. Eur. J. Pharm. Biopharm..

[44] Tsakok T., Woolf R., Smith C.H., Weidinger S., Flohr C. (2019). Atopic Dermatitis: The Skin Barrier and Beyond. Br. J. Dermatol..

[45] Lin T.-K., Crumrine D., Ackerman L.D., Santiago J.-L., Roelandt T., Uchida Y., Hupe M., Fabriàs G., Abad J.L., Rice R.H. (2012). Cellular Changes That Accompany Shedding of Human Corneocytes. J. Investig. Dermatol..

[46] Mauro T., Holleran W.M., Grayson S., Gao W.N., Man M.Q., Kriehuber E., Behne M., Feingold K.R., Elias P.M. (1998). Barrier Recovery Is Impeded at Neutral PH, Independent of Ionic Effects: Implications for Extracellular Lipid Processing. Arch. Dermatol. Res..

[47] Hachem J.-P., Roelandt T., Schürer N., Pu X., Fluhr J., Giddelo C., Man M.-Q., Crumrine D., Roseeuw D., Feingold K.R. (2010). Acute Acidification of Stratum Corneum Membrane Domains Using Polyhydroxyl Acids Improves Lipid Processing and Inhibits Degradation of Corneodesmosomes. J. Investig. Dermatol..

[48] Hachem J.-P., Crumrine D., Fluhr J., Brown B.E., Feingold K.R., Elias P.M. (2003). PH Directly Regulates Epidermal Permeability Barrier Homeostasis, and Stratum Corneum Integrity/Cohesion. J. Investig. Dermatol..

[49] Yamamoto T., Kurasawa M., Hattori T., Maeda T., Nakano H., Sasaki H. (2008). Relationship between Expression of Tight Junction-Related Molecules and Perturbed Epidermal Barrier Function in UVB-Irradiated Hairless Mice. Arch. Dermatol. Res..

[50] Kligman L.H., Murphy G.F. (1996). Ultraviolet B Radiation Increases Hairless Mouse Mast Cells in a Dose-Dependent Manner and Alters Distribution of UV-Induced Mast Cell Growth Factor. Photochem. Photobiol..

[51] Bosset S., Bonnet-Duquennoy M., Barré P., Chalon A., Kurfurst R., Bonté F., Schnébert S., Le Varlet B., Nicolas J.F. (2003). Photoageing Shows Histological Features of Chronic Skin Inflammation without Clinical and Molecular Abnormalities. Br. J. Dermatol..

[52] Coussens L.M., Raymond W.W., Bergers G., Laig-Webster M., Behrendtsen O., Werb Z., Caughey G.H., Hanahan D. (1999). Inflammatory Mast Cells Up-Regulate Angiogenesis during Squamous Epithelial Carcinogenesis. Genes Dev..

[53] Andreu P., Johansson M., Affara N.I., Pucci F., Tan T., Junankar S., Korets L., Lam J., Tawfik D., DeNardo D.G. (2010). FcRgamma Activation Regulates Inflammation-Associated Squamous Carcinogenesis. Cancer Cell.

[54] Chacón-Salinas R., Limón-Flores A.Y., Chávez-Blanco A.D., Gonzalez-Estrada A., Ullrich S.E. (2011). Mast Cell-Derived IL-10 Suppresses Germinal Center Formation by Affecting T Follicular Helper Cell Function. J. Immunol..

[55] Ashida Y., Denda M., Hirao T. (2001). Histamine H1 and H2 Receptor Antagonists Accelerate Skin Barrier Repair and Prevent Epidermal Hyperplasia Induced by Barrier Disruption in a Dry Environment. J. Investig. Dermatol..

[56] Albibas A.A., Rose-Zerilli M.J.J., Lai C., Pengelly R.J., Lockett G.A., Theaker J., Ennis S., Holloway J.W., Healy E. (2018). Subclonal Evolution of Cancer-Related Gene Mutations in P53 Immunopositive Patches in Human Skin. J. Investig. Dermatol..

[57] Haratake A., Uchida Y., Schmuth M., Tanno O., Yasuda R., Epstein J.H., Elias P.M., Holleran W.M. (1997). UVB-Induced Alterations in Permeability Barrier Function: Roles for Epidermal Hyperproliferation and Thymocyte-Mediated Response. J. Investig. Dermatol..

[58] Liou A., Elias P.M., Grunfeld C., Feingold K.R., Wood L.C. (1997). Amphiregulin and Nerve Growth Factor Expression Are Regulated by Barrier Status in Murine Epidermis. J. Investig. Dermatol..

[59] Darido C., Georgy S.R., Jane S.M. (2016). The Role of Barrier Genes in Epidermal Malignancy. Oncogene.

[60] Livak K.J., Schmittgen T.D. (2001). Analysis of Relative Gene Expression Data Using Real-Time Quantitative PCR and the 2(-Delta Delta C(T)) Method. Methods.

