# Supplementary material for: Characterization of Permeability Barrier Dysfunction in a Murine Model of Cutaneous Field Cancerization Following Chronic UV-B Irradiation: Implications for the Pathogenesis of Skin Cancer
Source: Cancers (Basel). 2021 Aug 4;13(16):3935. doi: 10.3390/cancers13163935 (PMC8394893; doi:10.3390/cancers13163935)
Supplement: Supplementary file 1 [file cancers-13-03935-s001.zip › cancers-1327871-supplementary.pdf]

cancers

MDPI

Supplementary Material: Characterization of Permeability Barrier Dysfunction in a Murine Model of Cutaneous Field Cancerization Following Chronic UV-B Irradiation: Implications for the Pathogenesis of Skin Cancer

Juan Luis Santiago, Jose Ramon Muñoz-Rodriguez, Miguel Angel de la Cruz-Morcillo, Clara Villar-Rodriguez, Lucia Gonzalez-Lopez, Carolina Aguado, Miriam Nuncia-Cantarero, Francisco Javier Redondo-Calvo, Jose Manuel Perez-Ortiz and Eva Maria Galan-Moya

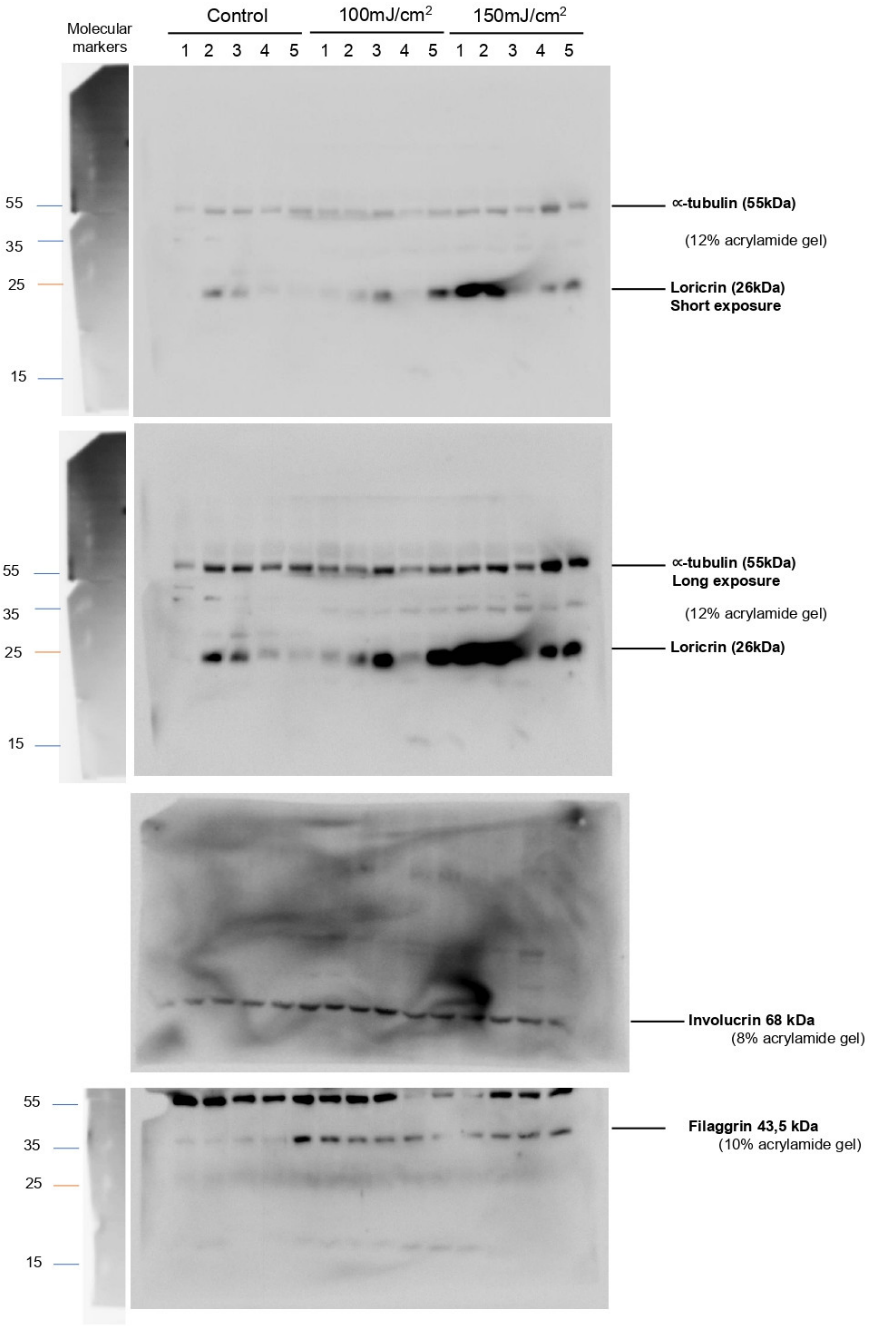

Figure S1. Uncropped original Westerm Blot.
